# Adrenalectomy for primary aldosteronism and its related surgical characteristics

**DOI:** 10.3389/fendo.2024.1416287

**Published:** 2024-06-20

**Authors:** Hao Xiang, Tingting Zhang, Wei Song, Deyong Yang, Xinqing Zhu

**Affiliations:** ^1^ Department of Urology, First Affiliated Hospital of Dalian Medical University, Dalian, China; ^2^ Department of Neurology, First Affiliated Hospital of Dalian Medical University, Dalian, China; ^3^ Department of Hypertension, First Affiliated Hospital of Dalian Medical University, Dalian, China; ^4^ Department of Surgery, Healinghands Clinic, Dalian, Liaoning, China

**Keywords:** adrenalectomy, primary hyperaldosteronism, hypertension, prognosis, CYP11B2

## Abstract

Primary aldosteronism (PA) is a common cause of secondary hypertension. Adrenalectomy is an effective treatment for unilateral PA, particularly aldosterone-producing adenoma (APA), resulting in improvements in biochemical parameters and blood pressure in the vast majority of patients. The article provides a comprehensive overview of PA, focusing on the outcomes of adrenalectomy for PA and the factors that may suggest prognostic implications. Analysis of the outcome of different PA patients undergoing adrenalectomy in terms of preoperative factors, vascular and adipose conditions, type of pathology, and somatic variants. In addition, it is recommended to use the histopathology of primary aldosteronism (HISTALDO) consensus to classify the patient’s pathological type, with classical and nonclassical pathological types showing a different prognosis and possibly being associated with an unresected contralateral adrenal gland. The primary aldosteronism surgical outcome (PASO) consensus sets uniform standards for postoperative outcomes in unilateral PA, but its setting of thresholds remains controversial. Partial adrenalectomy shows similar surgical results and fewer postoperative complications than total adrenalectomy, but there is a risk of missing the true source of abnormal aldosterone secretion. Steroid profiling and functional imaging techniques offer alternative options to adrenal vein sampling (AVS) for unilateral and bilateral judgments in patients with PA. A combination of factors is needed to predict the prognosis of PA patients undergoing adrenalectomy in order to manage patient expectations of the outcome of the procedure and to closely monitor blood pressure and biochemical parameters in patients who suggest a poorer prognosis.

## 1 Introduction

Primary aldosteronism (PA) was first described by Jerome W. Conn in 1954 and is characterized by hypokalemia and excessive production of aldosterone independent of the renin-angiotensin system (1). PA is the most common endocrine form of secondary hypertension. It has a prevalence of 5% to 15% in the general hypertensive population and up to 20% in those with severe or refractory hypertension (2, 3). Of these, approximately 27% have aldosterone-producing adenoma (APA) and 64% have bilateral adrenal hyperplasia (BAH) (4). The APA recommends unilateral adrenalectomy, while patients with BAH are treated with mineralocorticoid receptor antagonists.

## 2 Pathogenesis

Aldosterone is synthesized from cholesterol in the zona glomerulosa of the adrenal cortex by a variety of enzymes, the key step in the synthesis being aldosterone synthase (CYP11B2). PA mainly increases the gene transcription of CYP11B2 through various pathogenesis, and ultimately leads to increased aldosterone synthesis and cell proliferation. Based on the effect of gene mutate, somatic mutations are broadly categorized into three types: Ion Channels, Ion Transporters, and Cell Signaling Systems, which are discussed separately.

### 2.1 Ion channels (KCNJ5, CACNA1D, CACNA1H, CLCN2, SLC30A1)

KCNJ5 encodes a potassium inwardly rectifying channel (GIRK4), and mutations in KCNJ5 lead to channel selectivity changes that increase intracellular sodium influx, leading to cell depolarization (5). CACNA1D codes calcium voltage-gated channel subunit alpha1 D, CACNA1H codes calcium voltage-gated channel subunit alpha1 H, mutations in these genes lead to enhanced function of calcium channels and increased intracellular calcium concentration (6, 7). CLCN2 encodes chloride voltage-gated channel 2. The mutation resulted in enhanced chloride channel function and increased chloride ion permeability and depolarization (8). Rege, et al. (9) recently identified somatic mutations in the SLC30A1 gene in APAs. The SLC30A1 gene encodes the zinc transporter protein ZnT1. Mutations in SLC30A1 can potentially cause alterations in the cell membrane potential, which may impact the activity of voltage-gated calcium channels and consequently affect the influx of calcium ions and the regulation of intracellular calcium levels.

### 2.2 Ion transporters (ATP1A1, ATP2B3)

ATP1A1 encodes ATPase Na^+^/K^+^ transporting subunit alpha 1. The ATP1A1 mutate found in APAs leads to impaired potassium ion affinity and ATPase activity, leading to membrane depolarization. ATP2B encodes the ATPase plasma membrane Ca^2+^ transporting 3, which is responsible for pumping calcium ions inside the cell to the outside. Mutations in ATP2B3 affect the binding and transport of calcium ions, leading to the accumulation of calcium ions within the cell (10). Mutations in genes encoding ion channels or transporters ultimately result in an increase in intracellular Ca^2+^ concentration, activating a phosphorylation cascade that leads to increased aldosterone synthesis (11).

### 2.3 Cell signaling systems (GNAS, GNAQ, GNA11, PRKACA, CTNNB1, CADM1)

GNAS, GNAQ, and GNA1 encode G protein alpha subunits, and mutations in them can lead to abnormal activation of G protein signaling (12). PRKACA encodes protein kinase cAMP-activated catalytic subunit alpha. PRKACA mutates found in APAs lead to persistent activation of the cAMP/PKA signaling pathway, resulting in dysregulation of cell proliferation (13). Somatic mutate of the *CTNNB1* gene, which encodes a β-catenin, have been identified in APA, and the affected WNT/β-catenin signaling pathway is essential for the regulation of proliferation, differentiation and tumorigenesis in the adrenal cortex (14). However, the potential mechanism by which mutations in the *CTNNB1* gene lead to aldosterone overproduction is unclear.

In addition, somatic mutation of CADM1 was recently discovered in APAs, which is a synaptic cell adhesion molecule mainly expressed in the nervous system. The mutation of CADM1 leads to significant upregulation of CYP11B2 expression. This upregulation is associated with inhibition of intercellular communication, particularly by inhibiting communication at the gap junction (GJ) (15).

## 3 Diagnosis

The prevalence of hypertension combined with atrial fibrillation or diabetes mellitus was reported to be significantly higher in patients with PA than in those with essential hypertension (EHT) (16). Patients with PA also had a higher incidence of stroke than patients with EHT (12.9% v. s. 3.4%; 95% CI 2.0 to 8.6) (17). In addition, PA can lead to an increased risk of renal dysfunction and metabolic syndrome (18). Because even without considering the effect on blood pressure, aldosterone itself promotes cardiac and vascular fibrosis and tissue damage, leading to an increased incidence of cardiovascular and cerebrovascular events (11). The higher prevalence of diabetes in PA patients is mainly associated with subclinical hypercortisolism (SH) (19). Adequate treatment of PA can significantly reduce morbidity and mortality by reducing increased aldosterone and relieving renin suppression and hypertension (2). Early diagnosis and appropriate treatment of PA are therefore essential to reduce the increased risk associated with the disease.

The diagnosis of PA involves three stages: screening tests, case confirmation and classification of PA subtypes (20). Screening tests: Plasma aldosterone renin ratio (ARR), derived from measurement of plasma aldosterone concentrations (PACs) and plasma renin activity (PRA) or direct renin concentration (DRC), is the currently recommended screening method. In recent years, as ARR has been used in an increasing number of hypertensive patients, the detection of PA has increased significantly, especially in patients without hypokalemia (16). Confirmatory testing: The test was based on the assumption that aldosterone production would decrease if renin production were completely inhibited or if angiotensin II production was blocked. Common confirmatory tests include the fludrocortisone suppression test (FST), the saline infusion test (SIT), the oral salt loading test (SLT) and the captopril challenge test (CCT). FST has been used less frequently due to the need for hospitalization. SIT and SLT are the most commonly used in China and CCT is preferred for patients at risk of volume overload (16). Classification of PA subtypes: In most cases, PA is caused by either APA or BAH. The differential diagnosis between the two subtypes is important because the treatment of the two varies considerably. Masoni first introduced AVS in 1957 (21) and it has now become the gold standard for differentiating between unilateral and bilateral forms of PA. Conventional AVS collects blood samples from both central adrenal veins, identifying the laterality by comparison of steroid secretion, classifying PA subtypes, and usually guide for total adrenalectomy. In a multicenter study including 761 patients, unilateral PA patients diagnosed by AVS and subsequently treated by surgery have a higher rate of postoperative complete biochemical success than the CT group (22). In a study involving 19 centers and 1,625 patients, AVS-guided adrenalectomy patients had higher rates of hypertension cure than non-AVS-guided patients. The super-selective adrenal venous sampling, also known as segmental AVS (S-AVS), has been proposed providing a new basis for partial adrenalectomy. In contrast to central AVS (C-AVS), in addition to distinguishing between unilateral and bilateral diseases, S-AVS can assess intra-adrenal hormone distribution, pinpoint the site of aldosterone hypersecretion and make it possible for patients with PA treated by partial adrenalectomy (23).

However, as an invasive operation, AVS is technically difficult and needs to be performed in an experienced medical center. The overall complication rate for AVS is approximately 2.5%, the most common complication being groin hematoma and, in severe cases, adrenal hemorrhage and adrenal vein dissection (24).

## 4 Surgical treatment

**Figure 1:**
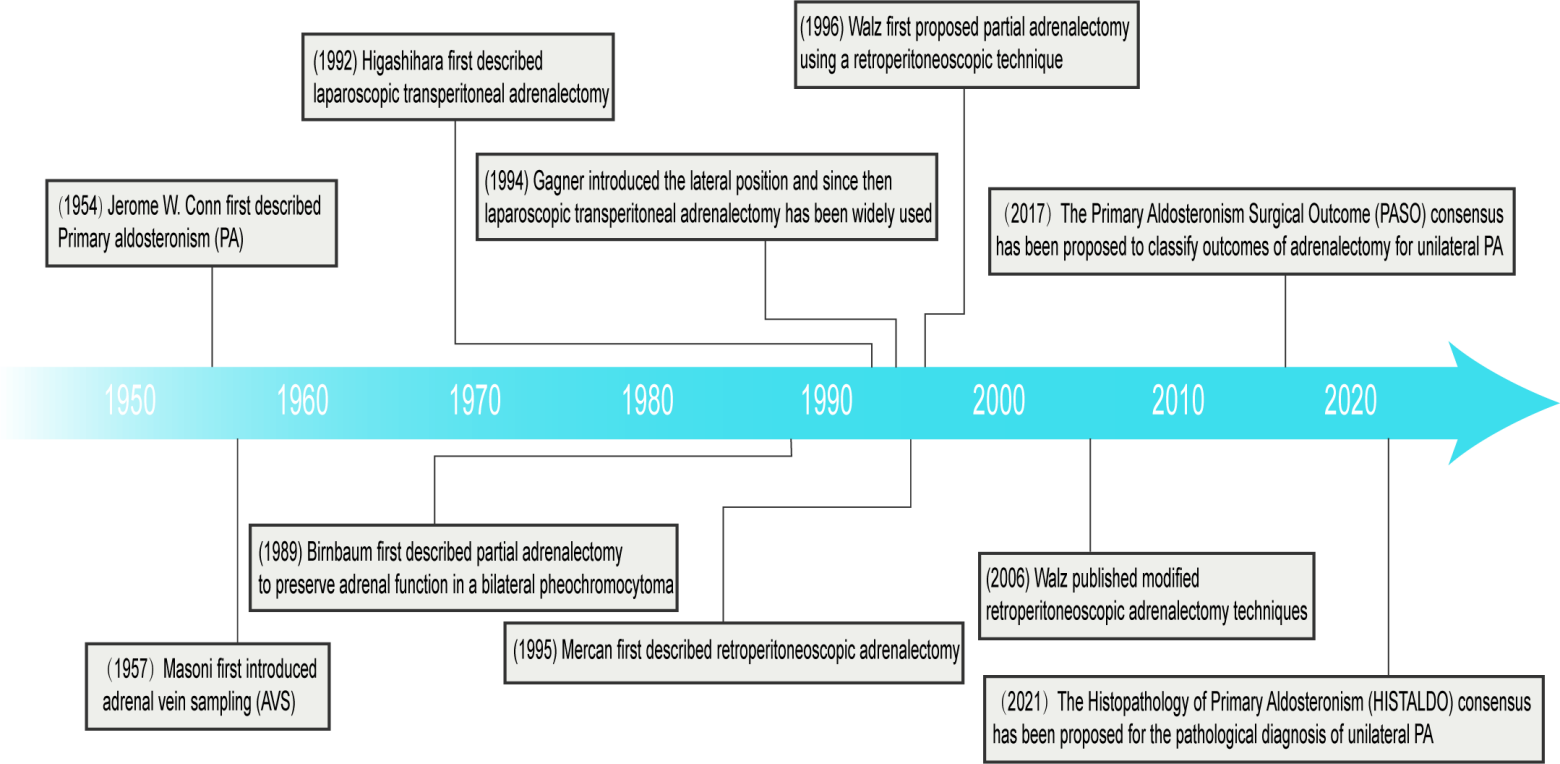
Milestones in primary aldosteronism and adrenalectomy.

Laparoscopic adrenalectomy is now a safe and effective standard surgical treatment option. Compared to traditional open surgery, laparoscopic adrenalectomy has shown significant advantages in terms of patient recovery and perioperative complications (25). Higashihara, et al. (26) first described laparoscopic transperitoneal adrenalectomy (LTA) in 1992 (**Figure 1**). Gagner, et al. (27) first introduced the lateral position in 1994 and since then LTA has been widely used. Mercan, et al. (28) first described retroperitoneoscopic adrenalectomy (RP) in 1995, but it was not routinely performed until the mid-2000s when Walz published modified techniques (29). In addition, Walz, et al. (30) first proposed partial adrenalectomy using a retroperitoneoscopic technique in 1996. The retroperitoneal approach has the advantage of not interfering the abdominal organs, avoiding intra-abdominal complications (e.g., postoperative intestinal obstruction, adhesions) and a shorter operative time, but the narrow space for the retroperitoneal approach makes it unsuitable for patients with large tumor diameters or poor periadrenal fat conditions (31). At the same time, all processes can be carried out with the assistance of a robot. Morino, et al. (32) compared the feasibility and safety of robot-assisted adrenalectomy (n = 10) with laparoscopic adrenalectomy (n = 10), and robot-assisted adrenalectomy did not show a significant advantage; instead, the robotic group had a longer operative time (*p* < 0.001), a higher perioperative complication rate (20% vs 0%), and a higher operative cost ($3,467 vs $2,737; *p* < 0.01). Therefore, further research is needed to fully define the role of robotic-assisted adrenalectomy in adrenalectomy (33).

## 5 Assessment of surgical outcomes

There is considerable variation between studies in the outcome of adrenalectomy in patients with PA, mainly due to differences in the definition of clinical success, resulting in considerable heterogeneity in prognosis (2). Williams, et al. (34) presented the Primary Aldosteronism Surgical Outcome (PASO) in 2017 to establish a standardized set of international consensuses for clinical and biochemical outcomes in unilateral PA adrenalectomy. Consensus assesses clinical outcomes based on blood pressure and use of antihypertensive medication, and biochemical outcomes based on blood potassium, ARR, and aldosterone concentrations. Clinical and biochemical outcomes were categorized as complete, partial and absent success (**Table 1**). They evaluated 705 patients with unilateral PA undergoing adrenalectomy at 12 centers from 1994 to 2015 using the PASO consensus. 259 (37%) of the 705 patients had complete clinical success and 334 (47%) had partial clinical success, i.e., over 80% of patients had improved blood pressure control. In addition, 656 of 699 patients (94%) had complete biochemical success (34).

**Table 1:** The Primary Aldosteronism Surgical Outcome (PASO).

Outcome	Clinical	Biochemical
Complete success	Normal blood pressure, no need for antihypertensive medication	Correction of hypokalemia (if present preoperatively) and normalization of ARR; in patients with high postoperative ARR, suppression of aldosterone secretion should be performed in the confirmatory test
Partial success	Same blood pressure as before surgery with less antihypertensive medication; or lower blood pressure with the same or less medication	Correction of hypokalemia (if present preoperatively); patients with elevated ARR who have more than 50% reduction in baseline plasma aldosterone concentration compared to preoperative or who have abnormal but improved postoperative confirmatory test results
Absent success	No change or increase in blood pressure, no change or increase in use of antihypertensive medication	Persistent hypokalemia (if preoperative) or persistent elevated ARR or both, failure of postoperative confirmatory tests to suppress aldosterone secretion

An initial postoperative outcome assessment of at least blood pressure and serum potassium concentration should be performed within the first 3 months to adjust antihypertensive medication and correct hyperkalemia/hyperkalemia if necessary. However, final results should be assessed at 6-12 months and reassessed annually after that.

Sawyer, et al. (35) conducted follow-up evaluations on 47 Australian PA patients who underwent unilateral adrenalectomy using the PASO criteria. The results showed that among the 40 patients who achieved clinical outcomes, 35.0% (14/40) had complete clinical success, and 47.5% (19/40) had partial clinical success. Among the 30 patients who achieved biochemical outcomes, 83.8% (31/37) had complete biochemical success. A total of 93.6% (44/47) of patients benefited from adrenalectomy. Similarly, Anceschi, et al. (36) assessed 90 PA patients who underwent unilateral adrenalectomy using the PASO criteria. Sixty-one patients underwent minimally-invasive total adrenalectomy, with 54% (33/61) achieving complete clinical success and 23% (14/61) achieving partial clinical success. Additionally, 81.9% (50/61) achieved complete biochemical success.

## 6 Factors affecting prognosis

Although the majority of patients with unilateral PA treated with adrenalectomy have significantly improved clinical and biochemical outcomes, some patients still have persistent postoperative hypertension or abnormal biochemical parameters. It is therefore hoped that an analysis of the likely prognosis of patients in terms of preoperative factors, vascular and adipose conditions, postoperative pathology and somatic cell variation will help manage patient expectations of postoperative outcomes and identify patients who require close follow-up or ongoing monitoring of blood pressure and biochemical parameters (3).

### 6.1 Preoperative factors

Predicting patient prognosis through preoperative factors also facilitates the selection of an appropriate treatment strategy for patients, particularly in patients at high risk for surgery and in patients with imaging of adrenal nodules for whom conclusive evidence of lateralized aldosterone excess is not available (37). In a retrospective study of 96 patients undergoing laparoscopic adrenalectomy for unilateral PA, Bokuda, et al. (38) concluded that BMI (*p* = 0.0473) and contralateral ratio (*p* = 0.0199) were significantly associated with normal postoperative blood pressure and no need for antihypertensive medication by multivariate logistic regression (**Table 2**).

**Table 2:** Preoperative factors affecting prognosis.

First author (year)	Design	Patients (n)	Characteristics	Outcome measure	Statistical analyses	Results
Williams (2017) (34)	Retrospective	unilateral PA Clinical data (n = 706) Biochemical data (n = 699)	Age, Sex, BMI Antihypertensive medication, Systolic blood pressure	PASO (complete plus partial clinical success)	Adjusted logistic regression analyses	Younger age (*p* = 0.004), female sex (*p* = 0.002), lower BMI (*p* = 0.001), higher systolic blood pressure (*p* = 0.005) and antihypertensive medication (*p* = 0.003) at baseline were determinants of clinical benefit.
Burrello (2020) (3)	Retrospective	unilateral PA (n = 380)	Duration of hypertension, Sex, BMI, AntiHT medication, Target organ damage, Largest nodule at imaging	PASO (complete clinical success)	Unadjusted univariate and adjusted multivariate logistic regression analyses	Duration of hypertension (*p* < 0.001), Sex (*p* < 0.001), BMI (*p* < 0.001), AntiHT medication (*p* < 0.001), Target organ damage (*p* < 0.001), and largest nodule at imaging (*p* = 0.048) were confirmed as predictors.
Bokuda (2017) (38)	Retrospective	unilateral PA (n = 96)	Age, Sex, BMI Antihypertensive medication, UA, CR	12-month follow-up Cured: normotensive without drugs Not cured: not normotensive	Multivariate logistic regression analyses	Higher BMI (*p* = 0.0473) significantly correlated with not cured, while lower CR (*p* = 0.0199) significantly correlated with cured.
Rossi (2008) (37)	Prospectively	APA (n = 50)	BMI, Systolic BP, M/L, Known duration of HT	Cured Markedly Improved Mildly Improved	Backward stepwise multivariable logistic regression analysis	M/L (*p* = 0.038; OR: 0.5992, 95%CI: 0.3695 to 0.9718); Known duration of HT (*p* = 0.033; OR: 0.9812, 95%CI: 0.9642 to 0.9985).
Picado (2021) (39)	Retrospective	PA (n = 37)	Age, sex, race, ethnicity, preoperative aldosterone and renin level, tumor size, BMI, duration of hypertension, the number of blood pressure medications	PASO (absent clinical success)	Multivariate logistic regression analyses	BMI (*p* = 0.04; OR: 1.13, 95%CI: 1.01 to 1.29); duration of hypertension (*p* < 0.05; OR: 1.11, 95%CI: 1.03 to 1.25); the number of blood pressure medications (*p* < 0.05; OR: 2.30, 95%CI: 1.07 to 4.93) were associated with absent clinical success.

AntiHT medication, antihypertensive medication; APA, aldosterone-­producing adenoma; BMI, body mass index; BP, blood pressure; CR, contralateral ratio; HT, hypertension; M/L, media: lumen ratio; PA, primary aldosteronism; PASO, primary aldosteronism surgical outcome; UA, uric acid.

Williams, et al. (34) used logistic regression analysis to identify preoperative factors associated with clinical and biochemical outcomes following the establishment of the PASO consensus, suggesting that younger and female patients were more likely to have complete clinical success or clinical improvement (complete + partial clinical success), while preoperative antihypertensive medication use and left ventricular hypertrophy were negatively associated with complete clinical success. Similarly, Picado, et al. (39) used the PASO consensus to assess long-term outcomes in 37 patients with PA who underwent adrenalectomy and to identify preoperative predictors associated with persistent postoperative hypertension. The results showed complete biochemical success in all patients, while clinical outcomes were complete success 15 (41%), partial success 14 (38%) and absent success 8 (21%). Multivariate logistic regression analysis showed that BMI (*p* = 0.04), duration of hypertension (*p* < 0.05) and the number of antihypertensive drugs used (*p* < 0.05) were significantly associated with absent clinical success.

Burrello, et al. (3) developed a 25-point scoring system using preoperative factors to predict clinical outcomes after unilateral PA. Data from 380 patients undergoing adrenalectomy for unilateral PA were first analyzed by unadjusted and adjusted logistic regression to select variables associated with clinical complete success, followed by the training and testing of linear discriminant analysis models to establish scores based on data from these 380 patients. A total of six variables were screened for the study: “duration of hypertension,” “sex,” “body mass index (BMI),” “antihypertensive medication,” “target organ damage” and “largest nodule at imaging.” Of these, duration of hypertension (negative correlation) was the strongest predictor of clinical complete success, followed by anti-hypertensive medication (negative correlation) and largest nodule at imaging (positive correlation). Each variable is assigned a different score, with higher total scores suggesting a better prognosis. Using a score of 16 as a cut-off value results in an accuracy of 79.2%, with sensitivities and specificities of 71.3% and 84.4% respectively.

### 6.2 Variations in the anatomy of the adrenal veins

**Figure 2:**
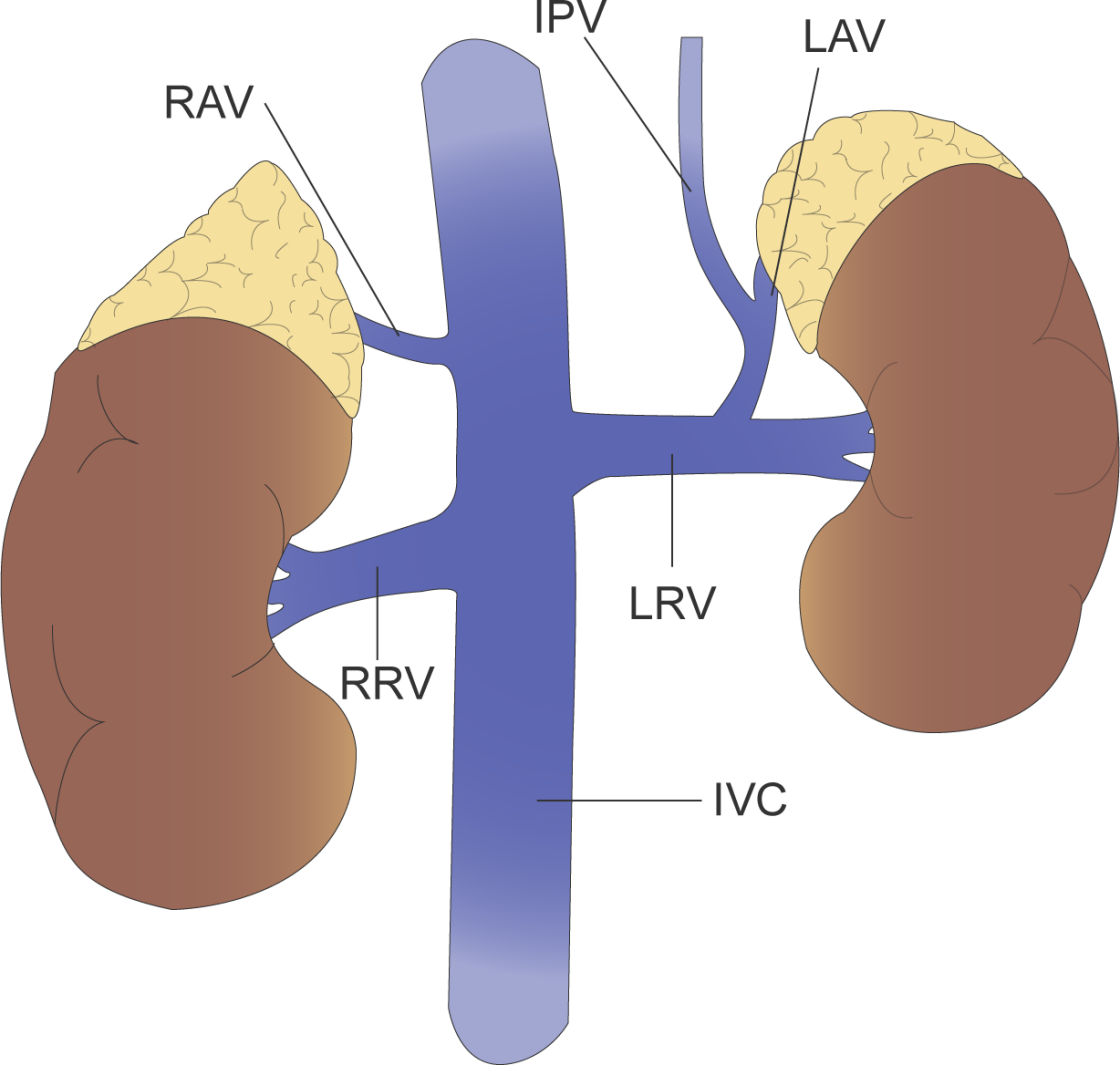
The left adrenal vein (LAV) receives inferior phrenic vein (IPV) and drains into left renal vein (LRV), while the right adrenal vein (RAV) drains directly into the inferior vena cava (IVC).

Management of the central adrenal vein is a key step in adrenalectomy and can lead to hemorrhage if not handled correctly (25). In addition, there may be variants of the adrenal vasculature, so a thorough knowledge of adrenal vein anatomy by the operator is required to avoid medically induced injury. The most common anatomy of the adrenal veins is that the left adrenal vein receives inferior phrenic and drains into left renal vein, while the right adrenal vein drains directly into the inferior vena cava (**Figure 2**).

**Figure 3:**
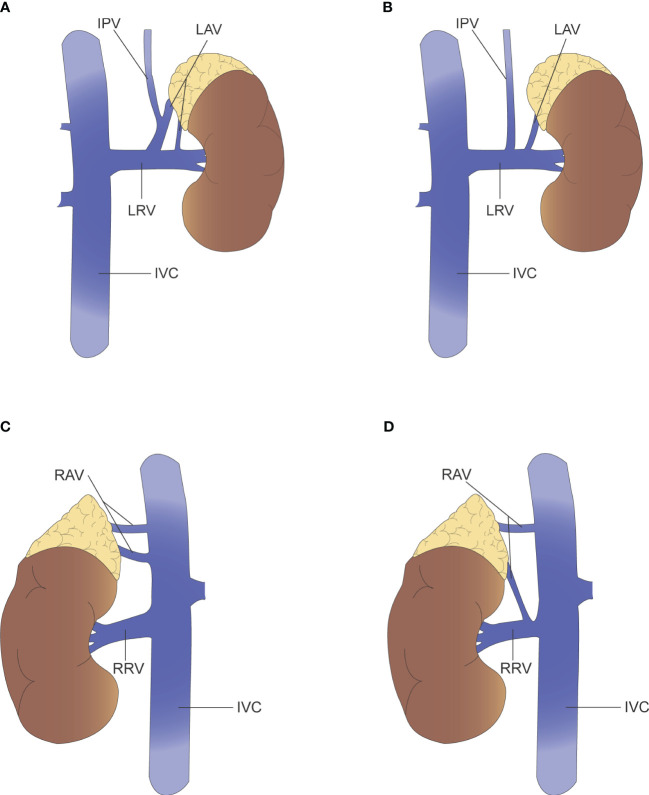
**(A)** Left renal vein receives adrenal vein and inferior phrenic vein common trunk and an accessory adrenal vein. **(B)** Left adrenal vein joins renal vein alone, renal vein receives independent inferior phrenic vein. **(C)** Two right adrenal vein and all drain into the IVC. **(D)** Two right adrenal vein, where one drains into the IVC and the other into the renal vein.

Cesmebasi, et al. (40) argue that the variations in adrenal venous drainage cannot be described independently, but rather the overall appearance of the adrenal veins and their accompanying renal veins are described in a unified manner. For example, the anatomical variation of the left adrenal vein is described as “Adrenal vein joins renal alone, renal vein receives independent inferior phrenic vein,” and “Double adrenal veins, one receives inferior phrenic vein, renal vein receives adrenal vein and inferior phrenic vein common trunk and an accessory adrenal vein (**Figure 3**).” Given the proximity of the right adrenal vein to the inferior vena cava and the variability of the right adrenal vein, it is recommended that special attention be paid to venous dissection during right adrenalectomy or AVS operations. Scholten, et al. (41) suggested that adrenal vein anatomical variation could be described in terms of both number and location. Of the 546 laparoscopic adrenalectomies collected, 70 (13%) had variations in adrenal vein anatomy. 63 had variation in the number of veins, including “no identifiable central adrenal vein,” “one central adrenal vein with additional prominent small veins,” and “multiple adrenal veins with or without small veins.” Seven cases were locational variations related to the hepatic vein, the inferior vena cava or the inferior phrenic vein. For instance, “the right adrenal vein joins the right hepatic vein.” Patients with variant venous anatomy had larger tumors (5.1 vs 3.3 cm; *p* < 0.01) and a higher proportion of pheochromocytomas (24 (35%) vs 100 (21%); *p* = 0.02) compared to patients with normal venous anatomy. The mean operative time was longer in patients with venous variants (*p* < 0.05) and the estimated blood loss (EBL) was also higher (*p* = 0.01). It was also found that more venous variants occurred on the right side than on the left (42 (17%) vs 28 (9%); *p* = 0.02), so the risk of medically induced injury during surgery was greater on the right side. Sun, et al. (42) reached a similar conclusion. In a retrospective analysis of 303 adrenalectomies, 62 cases (20%) had adrenal vein variation. Multiple logistic regression analysis showed that tumor size and pheochromocytoma were independent factors associated with variant veins. Multiple linear regression modeling of bleeding showed an increase of approximately 42.5% in patients with variant veins compared to normal veins (*p* = 0.009). In addition, patients with adrenal vein variants had more blood loss (*p* < 0.001), longer operative time (*p* < 0.001), longer postoperative hospital stay (*p* = 0.004) and higher operative costs (*p* = 0.014) compared to normal anatomy. Unfortunately, there is a lack of studies comparing variant adrenal venous anatomy with normal venous anatomy for long-term outcome after adrenalectomy.

It is important to note that in AVS procedures, although very rare, the use of unsuitable catheters and catheterization techniques may result in serious complications such as adrenal vein dissection due to inadequate knowledge of adrenal vein anatomy (43). In addition, even if the central vein is successfully cannulated, variant venous drainage of an APA may lead to misinterpretation of the results and even a “double-down” AVS result (bilateral adrenal suppression) (44). A thorough understanding of the normal anatomy of the adrenal vein and its many variants is therefore essential to avoid complications and medically induced injuries during procedures such as AVS and adrenalectomy.

### 6.3 Periadrenal adipose tissue

The adrenal glands are located in the retroperitoneal space above the kidneys and are surrounded by periadrenal adipose tissue. The increased amount of periadrenal adipose tissue increases the operational difficulty of laparoscopic surgery and the difficulty of dissecting anatomical landmarks such as the inferior vena cava and adrenal veins, prolonging the operative time and increasing the incidence of postoperative complications. Although BMI is the most commonly used anthropometric measure to assess obesity, it does not always accurately reflect the extent of visceral fat in patients (45). Lindeman, et al. (29) introduced the concept of the posterior adiposity index (PAI), which is the sum of the distance from the skin to the Gerota fascia (S-GF) and the perirenal fat distance (PNF), i.e., the distance from the skin to the renal parenchyma. In a multifactorial regression analysis of predictors of operative time in 56 patients undergoing retroperitoneoscopic adrenalectomy, PAI (PAI ≥ 9; *p* = 0.02) predicted increased operative time and morbidly obese patients significantly increased the challenge of retroperitoneoscopic surgery (**Table 3**). Pearlstein, et al. (46) explored the predictors of operative time for retroperitoneoscopic adrenalectomy over BMI, with periadrenal fat volume being an independent predictor of increased operative time in both univariate and multivariate analyses (both *p* < 0.01). However, PAI was a significant predictor of operative time in the univariate analysis (*p* < 0.01) but not statistically significant in the multivariate analysis (*p* = 0.81). They concluded that BMI per se did not affect operative time when controlling for variables such as periadrenal fat volume and left-right side of surgery. In contrast to Pearlstein’s report that periadrenal volume including adrenal lesions was an independent predictor of prolonged operative time for retroperitoneoscopic adrenalectomy, Rah, et al. (47) directly measured and analyzed the volume of periadrenal fat excluding the adrenal mass. Multiple regression analysis showed that both PAI (*p* = 0.027) and periadrenal fat volume (*p* = 0.024) were predictors of longer operative time, while BMI was not statistically significant (*p* = 0.239). However, after grouping based on the learning curve, periadrenal fat volume was an independent predictor of prolonged operative time only before the learning curve (*p* = 0.009). After the learning curve, the difficulties posed by periadrenal fat would be overcome *(p* = 0.054). It is important to emphasize that although adipose tissue may extend the duration of surgery, it is not significantly associated with estimated blood loss (EBL) and does not apparently influence a negative surgical outcome (29).

**Table 3:** Assessment of the periadrenal adipose tissue.

First author (year)	Design	Patients (n)	Characteristics	Outcome measure	Statistical analyses	Results
Lindeman (2019) (29)	Retrospective	LA (n = 57) RP (n = 56)	PAI Lesion size Side	Operative time Estimated blood loss	Multivariable linear regression analyses	Increasing PAI (*p* = 0.02), larger lesions (*p* = 0.01) and right site (*p* = 0.03) were predictive of longer operative time in RP; Nothing was significantly associated with estimated blood loss.
Pearlstein (2020) (46)	Retrospective	RP (n = 83)	Periadrenal fat volume Side Order of operation	Operative time	Multivariable linear random effects model	Periadrenal volume (*p* < 0.01), side (*p* < 0.01) and order of operation (*p* = 0.02) retained significance.
Rah (2021) (47)	Retrospective	RP (n = 284)	Depth of descended adrenal tumor location to kidney PAI Periadrenal fat volume Sex, Side, Surgeon, Diagnosis	Operative time	Multivariate logistic regression	Depth of descended adrenal tumor location to kidney (*p* = 0.002), PAI (*p* = 0.027), large periadrenal fat volume (*p* = 0.024), male (*p* = 0.012), right site (*p* = 0.031), surgeon A (*p* = 0.002) and pheochromocytoma (*p* = 0.003) were predictive of longer operative time.
Er (2020) (48)	Retrospective	APA (n = 100) EH (n = 41)	VFA Duration of hypertension	PASO	Logistic regression analysis	APA patients had smaller VFA (*p* = 0.021) than EH patents; smaller Log VFA (*p* < 0.001) and shorter duration of hypertension to PA diagnosis (*p* = 0.011) could independently predict the cure of hypertension.

APA, aldosterone-­producing adenoma; BMI, body mass index; EH, essential hypertension; LA, laparoscopic transabdominal adrenalectomy; PAI, posterior adiposity index; PASO, primary aldosteronism surgical outcome; RP, retroperitoneoscopic adrenalectomy; VFA, visceral fat area.

Er, et al. (48) examined the relationship between visceral adipose tissue and postoperative clinical outcomes in patients with PA. One hundred patients with APA who underwent adrenalectomy and 41 control patients with primary hypertension were included in the study. The visceral fat area (VFA) of each patient was measured by CT and showed that patients with PA had a significantly smaller VFA than patients with essential hypertension (*p* = 0.021). Logistic regression analysis showed that a smaller VAF (*p* < 0.001) and shorter duration of hypertension (*p* = 0.011) predicted complete clinical success after adrenalectomy. The reason for this may be that patients with a larger VAF are associated with obesity-related hypertension and do not fully normalize their blood pressure after undergoing adrenalectomy.

### 6.4 Type of pathology

Hematoxylin-eosin (HE) staining, routinely performed in the pathology laboratory, provides only morphological information, but it is not suitable for functional histopathological analysis and cannot determine the source of excess aldosterone (14). Immunohistochemical staining for CYP11B2, a key enzyme involved in aldosterone synthesis present in the zona glomerulosa, is important for the diagnosis of potential sources of excess aldosterone production and pathology in PA. The proposal of the international consensus on Histopathology of Primary Aldosteronism (HISTALDO), based on CYP11B2 immunohistochemical staining for classification and diagnosis, contributed to the standardized nomenclature of resected PA pathology and the consistency of histopathological diagnosis of unilateral PA (49). Consensus groups lesions into five categories: Aldosterone-producing adenoma (APA), Aldosterone-producing nodule (APN), Aldosterone-producing micronodule (APM) (previously known as aldosterone-producing cell clusters), Multiple aldosterone-producing nodules or multiple aldosterone-producing micronodules (MAPN or MAPM) (previously known as nodular hyperplasia or micronodular hyperplasia) and Aldosterone-producing diffuse hyperplasia (APDH). Of these, APA and APN are classified as classical unilateral primary aldosteronism and the others as nonclassical unilateral primary aldosteronism.

Williams, et al. (49) compared the classical group (n = 24) with the nonclassical group (n = 12). At baseline in the nonclassical group, hypertension lasted longer (*p* = 0.01), pathological nodules were smaller (*p* = 0.019), the lateralization index was lower (*p* = 0.048), and serum potassium concentrations were higher (*p* = 0.031); however, during postoperative follow-up, the nonclassical group showed lower serum potassium concentrations (*p* = 0.006) and a higher ARR (*p* = 0.006); according to the PASO criteria, although there were no statistical differences in clinical outcomes between the two groups, the biochemical results were worse in the nonclassical group than in the classical group (*p* = 0.009). Nanba, et al. (50) divided 32 patients undergoing unilateral adrenalectomy for PA into an APA group (n = 22) and a non-APA group (n = 10). The preoperative APA group had lower serum potassium concentrations (*p* < 0.05), a higher prevalence of hypokalaemia (*p* < 0.01) and a higher ARR (*p* < 0.01) than the non-APA group. Similarly, Meyer, et al. (51) divided 60 patients with unilateral PA who underwent adrenalectomy into classical group (n = 45) and nonclassical group (n = 15). Classical group exhibited higher plasma aldosterone concentrations (*p* = 0.008) and ARR (*p* = 0.002) at baseline level. In addition, the classical group had a significantly higher proportion of complete biochemical success (97.6% vs 66.7%, *p* = 0.004). These results suggest that PA patients in the classical group had more severe preoperative biochemical indicators, but had a better prognosis after undergoing adrenalectomy than PA patients in the nonclassical group.

In addition to CYP11B2 immunohistochemical staining, chemokine receptors CXCR4 immunohistochemical staining was also characteristically expressed. Heinze, et al. (52) found that CXCR4 showed strong staining in the subcapsular region of normal adrenal glands, as well as strong staining in APA and a significant positive correlation with CYP11B2 (*p* < 0.01), but was almost negative in non-functioning adenomas.

### 6.5 Somatic variants

Somatic variants of the *KCNJ5*, *CACNA1D*, *CACNA1H*, *CLCN2*, *ATP1A1*, *ATP2B3* and *CTNNB1* genes were found in unilateral PA (53). APA is the predominant lesion type in unilateral PA, with an overall somatic variant detection rate of approximately 50–58.4% (54–56). However, recent researchers have used immunohistochemistry to guide lesion selection, resulting in detectable somatic variation of up to 90% in APA (57). The dominant somatic variant in APA is a mutation in the *KCNJ5* gene, with an incidence of approximately 40% in studies from Western countries and an even higher incidence in studies from Asian countries, which can reach approximately 70%, but the *CACNA1D* variant is more common in African Americans, accounting for 42% (58).

Vilela, et al. (54) conducted a retrospective study of *KCNJ5* somatic variants associated with clinical outcomes in unilateral PA adrenalectomy, where *KCNJ5* somatic variants were identified in 33 (43.4%) of 76 patients who had their genes sequenced. When patients were divided into *KCNJ5* variant and wild-type groups for comparison, the proportion of complete clinical success was significantly higher in the variant group than in the wild-type group (57.6% vs 16.2%; *p* = 0.0001). Multiple logistic regression also showed that the *KCNJ5* somatic mutation was an independent predictor of complete clinical success (RR 6.418, 95% CI 1.83 to 22.93; *p* = 0.004). Interestingly, a higher proportion of patients in the *KCNJ5* variant group were female (*p* = 0.004) and the size of the tumor was larger (*p* = 0.001) compared to the wild-type group, which is consistent with the preoperative factors suggestive of a good prognosis for adrenalectomy described above (3, 34). Similar results were obtained by Kitamoto, et al. (59), where *KCNJ5* somatic variants were present in 106 (74.6%) of 142 patients with APA. 136 (95.8%) patients achieved complete endocrinological remission after adrenalectomy, with 81 (59.6%) patients cured of hypertension and 55 (40.4%) improved. When compared between the two groups, the proportion of *KCNJ5* somatic variants was significantly higher in the cured group (85.2% vs 60%; *p* = 0.002). Stepwise regression analysis also demonstrated that *KCNJ5* somatic variants, duration of hypertension and number of antihypertensive medications used were independently associated with postoperative hypertensive remission. Also, patients with the *KCNJ5* somatic variants were younger, had larger tumors, had a more severe PA phenotype and showed more aggressive disease progression than patients with the wild-type *KCNJ5* gene.

## 7 Discussion

### 7.1 Preoperative prognostic indicators that may influence surgical decisions

It has been suggested that persistent postoperative hypertension may be due to the diagnosis of highly asymmetric bilateral PA as unilateral PA during preoperative classification by AVS (51). In other words, the poor prognosis of unilateral adrenalectomy may have a strong association with the contralateral adrenal gland that was not removed. In a single-center prospective cohort study by Meyer, et al. (51), the biochemical outcomes were significantly better in the classical pathology type group than in the nonclassical group, while the ratio of absolute aldosterone concentration in the contralateral adrenal vein to the peripheral vein was significantly higher in the nonclassical group compared to the classical group (*p* = 0.004), with weaker contralateral suppression in this type of patient, asymmetric bilateral disease may have been present preoperatively. In another study, 43 patients with a biochemical outcome of absent + partial success compared with 52 patients with complete success, patients in the absent + partial success group exhibited a lower lateralization index and a higher contralateral ratio (60). Suggesting weaker contralateral adrenal suppression and abnormal postoperative aldosterone secretion on the contralateral side contributed to the inability to achieve a biochemical outcome of complete success. It has also been suggested that primary hypertension due to obesity, old age or long-term vascular damage and remodeling is likely to prevent patients from returning to normal blood pressure after adrenalectomy (39, 48). However, compared to the inability of mineralocorticoid antagonists to completely inhibit the systemic effects of aldosterone (atrial fibrillation, cardiac fibrosis), surgical treatment has a high biochemical success rate, allowing for the normalization of plasma aldosterone concentrations and providing better long-term benefits (61).

### 7.2 Histological/genetic features that may influence long-term prognosis

From a pathogenetic point of view, the prognosis of nonclassical PA differs from that of classical PA, probably because of a different genetic profile (53). The *KCNJ5* gene variant is the most common in APA (59, 62), while the *CACNA1D* gene variant is more common in APM (63, 64), with different somatic variants leading to different degrees of symptoms and prognosis. Moreover, the proportion of somatic variants and pathological types varies widely by region and ethnicity, especially in Asia compared with Western countries, where a higher proportion of APA patients have *KCNJ5* gene variants and a lower proportion of nonclassical PA (49, 58, 65).

### 7.3 Application of PASO consensus

Previous studies on unilateral PA have lacked uniform criteria for postoperative assessment, making the results highly heterogeneous and difficult to compare between studies. The advent of the PASO consensus has provided norms for the clinical and biochemical assessment of unilateral PA patients after adrenalectomy, but there are still some shortcomings in the postoperative assessment of adrenalectomy. Vorselaars, et al. (66) conducted a multicenter retrospective study to classify the postoperative outcomes of 380 patients from 16 treatment centers using the PASO consensus. However, 11% and 47% (16% of the total cohort) of patients classified as partial and absent clinical success, respectively, were considered to be misclassified or debatable. The main reasons for the debatable grouping of results were the PASO consensus’s use of high thresholds to determine relevant changes in systolic blood pressure (SBP) and the use of percentages rather than absolute values to determine changes in the defined daily dose. The PASO consensus defines a change in SBP of more than 20 mmHg to be considered a change in blood pressure. However, a systematic review by Ettehad, et al. (67) demonstrated that a 10mmHg reduction in SBP in hypertensive patients reduced the risk of major cardiovascular events (20%), coronary heart disease (17%), stroke (27%), heart failure (28%) and all-cause mortality (13%). A cut-off value of 20 mmHg may allow a significant proportion of patients with partial clinical success to be judged as having absent clinical success. On the other hand, the consensus definition of reduction in antihypertensive medication use refers to a 50% or greater reduction in defined daily dose (DDD) between preoperative and postoperative periods. Considering only percentages without incorporating absolute changes in actual medication use may result in medication use reductions that are not clinically meaningful being judged as partial clinical success or substantial medication use reductions being judged as absent clinical success. In addition, the PASO lacks an assessment of surgical indicators. For example, the duration of surgery, estimated bleeding, and postoperative complications will significantly affect the patient’s recovery and quality of life, and the lack of these indicators does not facilitate the overall assessment of the outcome of the procedure (68).

### 7.4 New diagnostic method

CT and AVS are commonly used to differentiate between unilateral and bilateral PA, but CT has limitations in the diagnosis of adrenal lesions. It is unable to identify smaller APN or APM_S_ that are not morphologically distinct from the surrounding tissue, and even when a larger volume of tumor is observed, CT is unable to determine whether it is secretory or not. A systematic review of the diagnostic concordance of CT and MRI with AVS conducted by Kempers, et al. (69) concluded that of the 950 patients included in the 38 studies, 37.8% had CT/MRI findings that were inconsistent with AVS, 14.6% of patients with bilateral PA would undergo adrenalectomy based on CT/MRI findings, 19.1% of patients with unilateral PA could not undergo adrenalectomy, and even 3.9% of patients were diagnosed with the wrong side. The guidelines therefore recommend that AVS should be performed preoperatively in patients with PA who are being considered for surgery, except for young patients (<35 years) with significant aldosterone excess and spontaneous hypokalaemia and a typical unilateral cortical adenoma on CT of the adrenal lesion, who can be crossed over to AVS before adrenalectomy (70).

However, AVS is an invasive procedure that is difficult and complex and carries the risk of complications. Recently, steroid profiling of peripheral veins and functional imaging techniques have provided additional options for differentiating subtypes (16). In a multicenter study of steroid profiling in patients with PA, the subtype could be correctly identified in 172 (80%) of 216 patients with PA based on the analysis of 12 adrenal steroids measured in peripheral blood (71). In addition, steroid profiling can be applied to predict the biochemical outcome of patients after adrenalectomy.Meyer, et al. (60) measured 15 adrenal steroids in the peripheral veins of patients with PA. Of the 70 patients in whom the measurements were performed, biochemical outcomes following adrenalectomy and the diagnosis of bilateral PA could be correctly predicted in 53 (76%) patients using linear discriminant analysis, which further increased the accuracy to 86% using decision tree analysis. As for functional imaging techniques, Heinze, et al. (52) used 68Ga-pentixafor-PET (selectively binds to human CXCR4) in nine patients with APA and found significantly higher tracer uptake on the side of increased aldosterone secretion (*p* < 0.01), which could effectively differentiate APA from non-functioning adenoma. However, more large RCT studies are needed to truly introduce these techniques into clinical practice.

### 7.5 Partial or total adrenalectomy

The concept of partial adrenalectomy was developed to treat hereditary and sporadic bilateral tumors, to reduce the risk of Addisonian crisis and to avoid the need for steroid replacement (72). Birnbaum, et al. (73) first described partial adrenalectomy to preserve adrenal function in a bilateral pheochromocytoma, and follow-up after 32 months showed that the patient had normal blood pressure and did not require antihypertensive medication or steroid replacement. Walz, et al. (30) first proposed partial adrenalectomy using a retroperitoneoscopic technique in 1996 and performed subtotal resection in five cases of smaller eccentric tumors, demonstrating that with careful selection, endocrine cure could also be achieved in unilateral pheochromocytomas and Conn adenomas. In recent years, the use of minimally invasive adrenal-sparing techniques for PA has increased with increasing experience and the spread of robotic surgery. Theoretically, in multiple occupying lesions or nonclassical PA, micronodules in the residual tissue after partial adrenalectomy have an impact on clinical parameters (blood pressure, plasma renin activity, plasma aldosterone) and they may play a role in PA recurrence. However, a systematic review including four studies (two RCTs) showed no significant differences in clinical and biochemical outcomes and recurrence rates between partial and total adrenalectomy (**Table 4**) (75). In another systematic review of 60 studies of partial adrenalectomy, the recurrence rate of PA was only 2% and 97% of patients did not require steroid replacement (72). Anceschi, et al. (68) used PASO criteria to assess the outcome of partial adrenalectomy compared to total adrenalectomy and showed that the proportion of patients with complete clinical success was higher in the partial adrenalectomy group than in the total adrenalectomy group (72.4% vs 54.1%) and the success rate of partial clinical success was lower than in the total adrenalectomy group (6.8% vs 23%), but there were differences in the baseline characteristics (patients in the partial adrenalectomy group had a smaller mean tumor diameter) and the surgical approach (most patients in the partial adrenalectomy group were robotic) between the two groups. Billmann, et al. (74) evaluated 234 patients with unilateral PA, 78 (33.3%) underwent minimally invasive partial adrenalectomy and 156 (66.7%) underwent minimally invasive total adrenalectomy. In terms of postoperative morbidity, the incidence of hypocortisolism and hypoglycemia was lower with partial adrenalectomy.

**Table 4:** Partial adrenalectomy vs Total adrenalectomy in patients with primary aldosteronism.

First author (year)	Design	Patients (n)	Protocol	Outcome measure	Results	Follow-up
Billmann (2021) (74)	Retrospective	Partial (n = 78) Total (n = 156)	pMIA: the adrenal adenoma was well localized and could be differentiated from adrenal tissue;tMIA: other conditions	Primary outcome:peri- and postoperative complications Secondary outcomes: (1) clinical and biochemical success (2) persistence/recurrence of the disease (3) operation duration (4) hospital stays (5) blood loss.	(1) Perioperative complications were comparable between both groups; (2) Postoperative hypocortisolism: pMIA (11.5%) vs tMIA (25.0%) (*p* < 0.001); Postoperative hypoglycemia: pMIA (2.6%) vs tMIA (7.1%) (*p* = 0.039); (3) No significant difference could be found between the 2 groups in secondary outcomes; (4) No recurrence was encountered in either the pMIA or the tMIA group.	24 months
Anceschi (2021) (68)	Retrospective	MITA (n = 61) MIPA (n = 29)	MIPA were limited to small tumors (<3 cm)	PASO clinical success Perioperative outcomes	(1) MITA group was higher in the tumor size (4.2 vs 2.7; *p* = 0.001), MIPA group was higher in the preoperative hypertension rate (82.8% vs 57.4%; *p* = 0.01); (2) MIPA group was higher in the complete clinical success rate (72.4% vs 54.1%, *p* = 0.097), MITA group was higher in the partial clinical success (23% vs 6.8%, *p* = 0.136); (3) LOS was increased in the MITA group (4 vs 3 d, *p* = 0.038);The perioperative transfusion rate, 24 h △Hb and overall complications were similar between groups.	42 months
Muth (2015) (75)	Systematic review	2RCTs, Prospective, Retrospective (n = 535)	Total ADX (n = 329) Partial ADX (n = 206	Normalized ARR (%), Hypertension cured/improved (%), Normalized K^+^ (%)	No difference in ARR, BP and potassium values improvement between patients randomized to partial or total adrenalectomy.	0.5-5.2 years

ADX, adrenalectomy; LOS, length of hospital stays; MIPA, minimally invasive partial adrenalectomy; MITA, minimally invasive total adrenalectomy; pMIA, partial minimally invasive adrenalectomy; PASO, primary aldosteronism surgical outcome; RCTs, randomized clinical trials; tMIA, total minimally invasive adrenalectomy.

Although the above findings suggest that partial adrenalectomy has similar surgical outcomes to total adrenalectomy and even fewer postoperative complications. However, partial adrenalectomy still has the potential to miss a true source of abnormal aldosterone. Nanba, et al. (50) used CYP11B2 immunostaining to classify postoperative pathology in patients with PA. Preoperative CT in 23 patients showed unilateral adrenal tumors, but in four (17.4%) of these patients the tumors did not show positive CYP11B2 immunostaining, suggesting that the tumors shown on CT may not be the true source of abnormal aldosterone secretion. In a case report by Ito, et al. (76), the patient had a preoperative diagnosis of right-sided PA by CT and AVS, etc. Postoperative pathology revealed multiple nodules (up to 6 mm) and hyperplasia of the zona glomerulosa by visual observation, but all these nodules were negative for CYP11B2 immunostaining, while 1 mm-sized micronodule positive for CYP11B2 immunostaining were found at other sites. The authors also encountered a case of a patient with unilateral PA who, 10 years after undergoing partial adrenalectomy on the right side, developed a recurrence in the ipsilateral adrenal gland, rediscovered APA, and underwent total adrenalectomy.Therefore, more large RCT studies and especially long- term follow-up are still needed to verify which surgical approach is of greater benefit.

## 8 Conclusion

PA is the most common endocrine form of secondary hypertension. Adrenalectomy for unilateral PA is effective. The patient’s preoperative factors, vascular and adipose conditions, type of pathology and somatic variants all suggest prognosis to varying degrees. Combining the indicators for analysis can better help the operator manage the patient’s prognostic expectations and target patients with potentially poorer prognoses for close monitoring of blood pressure and biochemical indicators. The emergence of the PASO consensus has set a uniform standard for the assessment of surgical outcomes in patients undergoing adrenalectomy for PA, but improvements are still needed. The use of CYP11B2 immunostaining for pathological diagnosis, as advocated by the HISTALDO consensus, can help to better identify potential sources of abnormal aldosterone secretion. Steroid profiling and functional imaging techniques offer new options for determining subtypes of PA as less invasive screening techniques. A combination of techniques and indicators allows for better early diagnosis of PA, better determination of the type of lesion and the selection of the appropriate surgical approach for timely surgical intervention in patients with unilateral PA.

## Author contributions

HX: Writing – original draft, Visualization, Software, Methodology, Data curation, Conceptualization. TZ: Writing – original draft, Visualization, Data curation, Conceptualization. WS: Writing – review & editing, Validation, Conceptualization. DY: Writing – review & editing, Visualization, Validation, Methodology, Conceptualization. XZ: Writing – review & editing, Visualization, Validation, Methodology, Conceptualization.

## References

[1] Conn JW. Part I. Painting background. Part II. Primary aldosteronism, a new clinical syndrome, 1954. J Lab Clin Med. (1990) 116:253–67. 2203856

[2] Vorselaars WMCM Nell S Postma EL Zarnegar R Drake FT Duh Q-Y. Clinical outcomes after unilateral adrenalectomy for primary aldosteronism. JAMA Surg. (2019) 154(4):e185842. doi: 10.1001/jamasurg.2018.5842 30810749 PMC6484800

[3] Burrello J Burrello A Stowasser M Nishikawa T Quinkler M Prejbisz A. The primary aldosteronism surgical outcome score for the prediction of clinical outcomes after adrenalectomy for unilateral primary aldosteronism. Ann Surg. (2020) 272:1125–32. doi: 10.1097/sla.0000000000003200 30672800

[4] Monticone S Burrello J Tizzani D Bertello C Viola A Buffolo F. Prevalence and clinical manifestations of primary aldosteronism encountered in primary care practice. J Am Coll Cardiol. (2017) 69:1811–20. doi: 10.1016/j.jacc.2017.01.052 28385310

[5] Choi M Scholl UI Yue P Björklund P Zhao B Nelson-Williams C. K+ channel mutations in adrenal aldosterone-producing adenomas and hereditary hypertension. Sci (New York NY). (2011) 331:768–72. doi: 10.1126/science.1198785 PMC3371087 21311022

[6] Azizan EA Poulsen H Tuluc P Zhou J Clausen MV Lieb A. Somatic mutations in ATP1A1 and CACNA1D underlie a common subtype of adrenal hypertension. Nat Genet. (2013) 45:1055–60. doi: 10.1038/ng.2716 23913004

[7] Scholl UI Stölting G Nelson-Williams C Vichot AA Choi M Loring E. Recurrent gain of function mutation in calcium channel CACNA1H causes early-onset hypertension with primary aldosteronism. eLife. (2015) 4:e06315. doi: 10.7554/eLife.06315 25907736 PMC4408447

[8] Fernandes-Rosa FL Daniil G Orozco IJ Göppner C El Zein R Jain V. A gain-of-function mutation in the CLCN2 chloride channel gene causes primary aldosteronism. Nat Genet. (2018) 50:355–61. doi: 10.1038/s41588-018-0053-8 29403012

[9] Rege J Bandulik S Nanba K Kosmann C Blinder AR Plain A. Somatic SLC30A1 mutations altering zinc transporter ZnT1 cause aldosterone-producing adenomas and primary aldosteronism. Nat Genet. (2023) 55:1623–31. doi: 10.1038/s41588-023-01498-5 PMC12051258 37709865

[10] Beuschlein F Boulkroun S Osswald A Wieland T Nielsen HN Lichtenauer UD. Somatic mutations in ATP1A1 and ATP2B3 lead to aldosterone-producing adenomas and secondary hypertension. Nat Genet. (2013) 45:440–4, 44e1–2. doi: 10.1038/ng.2550 23416519

[11] Zennaro M-C Boulkroun S Fernandes-Rosa FL. Pathogenesis and treatment of primary aldosteronism. Nat Rev Endocrinol. (2020) 16:578–89. doi: 10.1038/s41574-020-0382-4 32724183

[12] Tetti M Gong S Veglio F Reincke M Williams TA. Primary aldosteronism: Pathophysiological mechanisms of cell death and proliferation. Front Endocrinol (Lausanne). (2022) 13:934326 [published Online First: 2022/08/26. doi: 10.3389/fendo.2022.934326 36004349 PMC9393369

[13] Rhayem Y Perez-Rivas LG Dietz A Bathon K Gebhard C Riester A. PRKACA somatic mutations are rare findings in aldosterone-producing adenomas. J Clin Endocrinol Metab. (2016) 101:3010–7. doi: 10.1210/jc.2016-1700 27270477

[14] Sun L Jiang Y Xie J Zhu H Wu L Zhong X. Immunohistochemical analysis of CYP11B2, CYP11B1 and beta-catenin helps subtyping and relates with clinical characteristics of unilateral primary aldosteronism. Front Mol Biosci. (2021) 8:751770 [published Online First: 2021/10/12. doi: 10.3389/fmolb.2021.751770 34631800 PMC8497787

[15] Wu X Azizan EAB Goodchild E Garg S Hagiyama M Cabrera CP. Somatic mutations of CADM1 in aldosterone-producing adenomas and gap junction-dependent regulation of aldosterone production. Nat Genet. (2023) 55:1009–21. doi: 10.1038/s41588-023-01403-0 PMC10260400 37291193

[16] Reincke M Bancos I Mulatero P Scholl UI Stowasser M Williams TA. Diagnosis and treatment of primary aldosteronism. Lancet Diabetes Endocrinol. (2021) 9:876–92. doi: 10.1016/s2213-8587(21)00210-2 34798068

[17] Milliez P Girerd X Plouin P-F Blacher J Safar ME Mourad J-J. Evidence for an increased rate of cardiovascular events in patients with primary aldosteronism. J Am Coll Cardiol. (2005) 45:1243–48. doi: 10.1016/j.jacc.2005.01.015 15837256

[18] Rossi G-P Sechi LA Giacchetti G Ronconi V Strazzullo P Funder JW. Primary aldosteronism: cardiovascular, renal and metabolic implications. Trends Endocrinol Metab. (2008) 19:88–90. doi: 10.1016/j.tem.2008.01.006 18314347

[19] Akehi Y Yanase T Motonaga R Umakoshi H Tsuiki M Takeda Y. High prevalence of diabetes in patients with primary aldosteronism (PA) associated with subclinical hypercortisolism and prediabetes more prevalent in bilateral than unilateral PA: A large, multicenter cohort study in Japan. Diabetes Care. (2019) 42:938–45. doi: 10.2337/dc18-1293 31010944

[20] Williams TA Reincke M. MANAGEMENT OF ENDOCRINE DISEASE: Diagnosis and management of primary aldosteronism: the Endocrine Society guideline 2016 revisited. Eur J Endocrinol. (2018) 179:R19–29. doi: 10.1530/eje-17-0990 29674485

[21] Masoni A. Catheterisation of the right adrenal vein in man. Acta Med Scand. (1957) 159:225–34. doi: 10.1111/j.0954-6820.1957.tb00129.x 13508090

[22] Williams TA Burrello J Sechi LA Fardella CE Matrozova J Adolf C. Computed tomography and adrenal venous sampling in the diagnosis of unilateral primary aldosteronism. Hypertension. (2018) 72:641–49. doi: 10.1161/hypertensionaha.118.11382 29987100

[23] Omura M Saito J Matsuzawa Y Nishikawa T. Supper-selective ACTH-stimulated adrenal vein sampling is necessary for detecting precisely functional state of various lesions in unilateral and bilateral adrenal disorders, inducing primary aldosteronism with subclinical Cushing’s syndrome. Endocr J. (2011) 58:919–20. doi: 10.1507/endocrj.ej11-0210 21908932

[24] Thiesmeyer JW Ullmann TM Stamatiou AT Limberg J Stefanova D Beninato T. Association of adrenal venous sampling with outcomes in primary aldosteronism for unilateral adenomas. JAMA Surg. (2021) 156(2):165–71. doi: 10.1001/jamasurg.2020.5011 PMC7643039 33146695

[25] Wang X Liu J Ji A Liu C Nahayo S Wang L. The safety and efficiency of retroperitoneal laparoscopic adrenalectomy via extra and intra perinephric fat approaches: a retrospective clinical study. BMC Surg. (2019) 19(1):198. doi: 10.1186/s12893-019-0648-8 31864326 PMC6925459

[26] Higashihara E Tanaka Y Horie S Aruga S Nutahara K Homma Y. [A case report of laparoscopic adrenalectomy]. Nihon Hinyokika Gakkai Zasshi. (1992) 83:1130–3. doi: 10.5980/jpnjurol1989.83.1130 1387181

[27] Gagner M Lacroix A Bolte E Pomp A. Laparoscopic adrenalectomy. The importance of a flank approach in the lateral decubitus position. Surg Endosc. (1994) 8(2):135–8. doi: 10.1007/BF00316627 8165486

[28] Mercan S Seven R Ozarmagan S Tezelman S. Endoscopic retroperitoneal adrenalectomy. Surgery. (1995) 118:1071–76. doi: 10.1016/s0039-6060(05)80116-3 7491525

[29] Lindeman B Gawande AA Moore FD Cho NL Doherty GM Nehs MA. The posterior adiposity index: A quantitative selection tool for adrenalectomy approach. J Surg Res. (2019) 233:26–31. doi: 10.1016/j.jss.2018.07.003 30502257

[30] Walz MK Peitgen K Hoermann R Giebler RM Mann K Eigler FW. Posterior retroperitoneoscopy as a new minimally invasive approach for adrenalectomy: results of 30 adrenalectomies in 27 patients. World J Surg. (1996) 20:769–74. doi: 10.1007/s002689900117 8678949

[31] Walz MK Alesina PF Wenger FA Deligiannis A Szuczik E Petersenn S. Posterior retroperitoneoscopic adrenalectomy—results of 560 procedures in 520 patients. Surgery. (2006) 140:943–50. doi: 10.1016/j.surg.2006.07.039 17188142

[32] Morino M Benincà G Giraudo G Del Genio GM Rebecchi F Garrone C. Robot-assisted vs laparoscopic adrenalectomy: a prospective randomized controlled trial. Surg Endosc. (2004) 18:1742–46. doi: 10.1007/s00464-004-9046-z 15809781

[33] Hupe MC Imkamp F Merseburger AS. Minimally invasive approaches to adrenal tumors: an up-to-date summary including patient position and port placement of laparoscopic, retroperitoneoscopic, robot-assisted, and single-site adrenalectomy. Curr Opin Urol. (2017) 27:56–61. doi: 10.1097/MOU.0000000000000339 27533502

[34] Williams TA Lenders JWM Mulatero P Burrello J Rottenkolber M Adolf C. Outcomes after adrenalectomy for unilateral primary aldosteronism: an international consensus on outcome measures and analysis of remission rates in an international cohort. Lancet Diabetes Endocrinol. (2017) 5:689–99. doi: 10.1016/s2213-8587(17)30135-3 PMC5572673 28576687

[35] Sawyer N Glendenning P Vasikaran SD Page MM van Schie G Wong SL. The Adrenal Vein Sampling Outcomes Study (AVOS): success rates following adrenalectomy for unilateral primary aldosteronism. Pathology. (2023) 55:531–37. doi: 10.1016/j.pathol.2023.02.002 37062662

[36] Anceschi U Tufano A Flammia RS Mormando M Fiori C Zappalà O. Clinical cure vs a novel trifecta system for evaluating long-term outcomes of minimally-invasive partial or total adrenalectomy for unilateral primary aldosteronism: results of a multicentric series. Cent Eur J Urol. (2022) 75:345–51. doi: 10.5173/ceju.2022.147 PMC9903164 36794029

[37] Rossi GP Bolognesi M Rizzoni D Seccia TM Piva A Porteri E. Vascular remodeling and duration of hypertension predict outcome of adrenalectomy in primary aldosteronism patients. Hypertension. (2008) 51:1366–71. doi: 10.1161/HYPERTENSIONAHA.108.111369 18347224

[38] Bokuda K Yatabe M Mizuguchi Y Niiyama M Seki Y Watanabe D. Body mass index and contralateral ratio predict outcome following unilateral adrenalectomy in primary aldosteronism. Hypertens Res. (2017) 40:988–93. doi: 10.1038/hr.2017.78 28978983

[39] Picado O Whitfield BW Khan ZF Jeraq M Farrá JC Lew JI. Long-term outcome success after operative treatment for primary aldosteronism. Surgery. (2021) 169:528–32. doi: 10.1016/j.surg.2020.07.046 32948336

[40] Cesmebasi A Du Plessis M Iannatuono M Shah S Tubbs RS Loukas M. A review of the anatomy and clinical significance of adrenal veins. Clin Anat. (2014) 27:1253–63. doi: 10.1002/ca.22374 24737134

[41] Scholten A Cisco RM Vriens MR Shen WT Duh QY. Variant adrenal venous anatomy in 546 laparoscopic adrenalectomies. JAMA Surg. (2013) 148:378–83. doi: 10.1001/jamasurg.2013.610 23715888

[42] Sun F Zhuo R Ma W He H Ye L Xu D. Retrospective analysis of variant venous anatomy in 303 laparoscopic adrenalectomies and its clinical implications. J Surg Oncol. (2019) 119:801–06. doi: 10.1002/jso.25364 30697735

[43] Kobayashi K Alkukhun L Rey E Salaskar A Acharya R. Adrenal vein sampling: tips and tricks. Radiographics: Rev Publ Radiol Soc North America Inc. (2024) 44:e230115. doi: 10.1148/rg.230115 38662586

[44] DePietro DM Fraker DL Wachtel H Cohen DL Trerotola SO. Double-down” Adrenal vein sampling results in patients with apparent bilateral aldosterone suppression: utility of repeat sampling including super-selective sampling. J Vasc Intervent Radiol: JVIR. (2021) 32:656–65. doi: 10.1016/j.jvir.2020.12.029 PMC13214840 33781686

[45] Erbil Y Barbaros U Sari S Agcaoglu O Salmaslioglu A Ozarmagan S. The effect of retroperitoneal fat mass on surgical outcomes in patients performing laparoscopic adrenalectomy: the effect of fat tissue in adrenalectomy. Surg Innov. (2010) 17:114–9. doi: 10.1177/1553350610365703 20504787

[46] Pearlstein SS Kuo JH Chabot JA Lee JA. Periadrenal volume is a better predictor of prolonged operative time in laparoscopic retroperitoneal adrenalectomy than BMI. World J Surg. (2020) 44:578–84. doi: 10.1007/s00268-019-05324-0 31820058

[47] Rah C-S Kim WW Lee Y-M Chung K-W Koh J-M Lee SH. New predictive factors for prolonged operation time of laparoscopic posterior retroperitoneal adrenalectomy; retrospective cohort study. Int J Surg. (2021) 94:106–13. doi: 10.1016/j.ijsu.2021.106113 34534705

[48] Er LK Lin M-C Tsai Y-C Hsiao J-K Yang C-Y Chang C-C. Association of visceral adiposity and clinical outcome among patients with aldosterone producing adenoma. BMJ Open Diabetes Res Care. (2020) 8(1):e001153. doi: 10.1136/bmjdrc-2019-001153 PMC7383952 32713841

[49] Williams TA Gomez-Sanchez CE Rainey WE Giordano TJ Lam AK Marker A. International histopathology consensus for unilateral primary aldosteronism. J Clin Endocrinol Metab. (2021) 106:42–54. doi: 10.1210/clinem/dgaa484 32717746 PMC7765663

[50] Nanba K Tsuiki M Sawai K Mukai K Nishimoto K Usui T. Histopathological diagnosis of primary aldosteronism using CYP11B2 immunohistochemistry. J Clin Endocrinol Metab. (2013) 98:1567–74. doi: 10.1210/jc.2012-3726 23443813

[51] Meyer LS Handgriff L Lim JS Udager AM Kinker I-S Ladurner R. Single-center prospective cohort study on the histopathology, genotype, and postsurgical outcomes of patients with primary aldosteronism. Hypertension. (2021) 78:738–46. doi: 10.1161/hypertensionaha.121.17348 34024122

[52] Heinze B Fuss CT Mulatero P Beuschlein F Reincke M Mustafa M. Targeting CXCR4 (CXC chemokine receptor type 4) for molecular imaging of aldosterone-producing adenoma. Hypertension. (2018) 71:317–25. doi: 10.1161/hypertensionaha.117.09975 29279316

[53] Santana LS Guimaraes AG Almeida MQ. Pathogenesis of primary aldosteronism: impact on clinical outcome. Front Endocrinol. (2022) 13:927669. doi: 10.3389/fendo.2022.927669 PMC9261097 35813615

[54] Vilela LAP Rassi-Cruz M Guimaraes AG Moises CCS Freitas TC Alencar NP. KCNJ5 somatic mutation is a predictor of hypertension remission after adrenalectomy for unilateral primary aldosteronism. J Clin Endocrinol Metab. (2019) 104:4695–702. doi: 10.1210/jc.2019-00531 31216002

[55] Fernandes-Rosa FL Williams TA Riester A Steichen O Beuschlein F Boulkroun S. Genetic spectrum and clinical correlates of somatic mutations in aldosterone-producing adenoma. Hypertension. (2014) 64:354–61. doi: 10.1161/HYPERTENSIONAHA.114.03419 24866132

[56] Wu VC Wang SM Chueh SJ Yang SY Huang KH Lin YH. The prevalence of CTNNB1 mutations in primary aldosteronism and consequences for clinical outcomes. Sci Rep. (2017) 7:39121. doi: 10.1038/srep39121 28102204 PMC5244399

[57] Williams TA Reincke M. Pathophysiology and histopathology of primary aldosteronism. Trends Endocrinol Metab. (2022) 33:36–49. doi: 10.1016/j.tem.2021.10.002 34743804

[58] Nanba K Rainey WE. GENETICS IN ENDOCRINOLOGY: Impact of race and sex on genetic causes of aldosterone-producing adenomas. Eur J Endocrinol. (2021) 185:R1–R11. doi: 10.1530/eje-21-0031 33900205 PMC8480207

[59] Kitamoto T Omura M Suematsu S Saito J Nishikawa T. KCNJ5 mutation as a predictor for resolution of hypertension after surgical treatment of aldosterone-producing adenoma. J Hypertens. (2018) 36:619–27. doi: 10.1097/HJH.0000000000001578 29016532

[60] Meyer LS Wang X Sušnik E Burrello J Burrello A Castellano I. Immunohistopathology and steroid profiles associated with biochemical outcomes after adrenalectomy for unilateral primary aldosteronism. Hypertension. (2018) 72:650–57. doi: 10.1161/hypertensionaha.118.11465 PMC6202235 30012870

[61] Catena C Colussi G Lapenna R Nadalini E Chiuch A Gianfagna P. Long-term cardiac effects of adrenalectomy or mineralocorticoid antagonists in patients with primary aldosteronism. Hypertension. (2007) 50:911–8. doi: 10.1161/hypertensionaha.107.095448 17893375

[62] Nanba K Omata K Else T Beck PCC Nanba AT Turcu AF. Targeted molecular characterization of aldosterone-producing adenomas in white Americans. J Clin Endocrinol Metab. (2018) 103:3869–76. doi: 10.1210/jc.2018-01004 PMC6179168 30085035

[63] Pauzi FA Azizan EA. Functional characteristic and significance of aldosterone-producing cell clusters in primary aldosteronism and age-related hypertension. Front Endocrinol. (2021) 12:631848. doi: 10.3389/fendo.2021.631848 PMC7982842 33763031

[64] Omata K Anand SK Hovelson DH Liu C-J Yamazaki Y Nakamura Y. Aldosterone-producing cell clusters frequently harbor somatic mutations and accumulate with age in normal adrenals. J Endocr Soc. (2017) 1:787–99. doi: 10.1210/js.2017-00134 PMC5686701 29264530

[65] Wang H Wang F Zhang Y Wen J Dong D Chang X. Surgical outcomes of aldosterone-producing adenoma on the basis of the histopathological findings. Front Endocrinol. (2021) 12:663096. doi: 10.3389/fendo.2021.663096 PMC8451176 34552553

[66] Vorselaars WMCM van Beek D-J Postma EL Spiering W Borel Rinkes IHM Valk GD. Clinical outcomes after surgery for primary aldosteronism: Evaluation of the PASO-investigators’ consensus criteria within a worldwide cohort of patients. Surgery. (2019) 166:61–8. doi: 10.1016/j.surg.2019.01.031 31053245

[67] Ettehad D Emdin CA Kiran A Anderson SG Callender T Emberson J. Blood pressure lowering for prevention of cardiovascular disease and death: a systematic review and meta-analysis. Lancet. (2016) 387:957–67. doi: 10.1016/s0140-6736(15)01225-8 26724178

[68] Anceschi U Tuderti G Fiori C Zappalà O Ferriero MC Brassetti A. Minimally invasive partial versus total adrenalectomy for the treatment of primary aldosteronism: results of a multicenter series according to the PASO criteria. Eur Urol Focus. (2021) 7:1418–23. doi: 10.1016/j.euf.2020.06.023 32660839

[69] Kempers MJ Lenders JW van Outheusden L van der Wilt GJ Schultze Kool LJ Hermus AR. Systematic review: diagnostic procedures to differentiate unilateral from bilateral adrenal abnormality in primary aldosteronism. Ann Intern Med. (2009) 151:329–37. doi: 10.7326/0003-4819-151-5-200909010-00007 19721021

[70] Funder JW Carey RM Mantero F Murad MH Reincke M Shibata H. The management of primary aldosteronism: case detection, diagnosis, and treatment: an endocrine society clinical practice guideline. J Clin Endocrinol Metab. (2016) 101:1889–916. doi: 10.1210/jc.2015-4061 26934393

[71] Eisenhofer G Dekkers T Peitzsch M Dietz AS Bidlingmaier M Treitl M. Mass spectrometry-based adrenal and peripheral venous steroid profiling for subtyping primary aldosteronism. Clin Chem. (2016) 62:514–24. doi: 10.1373/clinchem.2015.251199 26787761

[72] Nagaraja V Eslick GD Edirimanne S. Recurrence and functional outcomes of partial adrenalectomy: A systematic review and meta-analysis. Int J Surg. (2015) 16:7–13. doi: 10.1016/j.ijsu.2015.01.015 25681039

[73] Birnbaum J Giuliano A Van Herle AJ. Partial adrenalectomy for pheochromocytoma with maintenance of adrenocortical function. J Clin Endocrinol Metab. (1989) 69:1078–81. doi: 10.1210/jcem-69-5-1078 2793991

[74] Billmann F Billeter A Thomusch O Keck T El Shishtawi S Langan EA. Minimally invasive partial versus total adrenalectomy for unilateral primary hyperaldosteronism-a retrospective, multicenter matched-pair analysis using the new international consensus on outcome measures. Surgery. (2021) 169:1361–70. doi: 10.1016/j.surg.2020.09.005 33077201

[75] Muth A Ragnarsson O Johannsson G Wängberg B. Systematic review of surgery and outcomes in patients with primary aldosteronism. Br J Surg. (2015) 102:307–17. doi: 10.1002/bjs.9744 25605481

[76] Ito A Yamazaki Y Sasano H Matsubara D Fukushima N Tamba M. A case of primary aldosteronism caused by unilateral multiple adrenocortical micronodules presenting as muscle cramps at rest: The importance of functional histopathology for identifying a culprit lesion. Pathol Int. (2017) 67:214–21. doi: 10.1111/pin.12521 28261922

